# Traditional Knowledge and Efficacy Analysis of an Emerging Medicinal Food Plant: *Disporopsis aspersa*

**DOI:** 10.3390/foods14010072

**Published:** 2024-12-30

**Authors:** Qingyu Chen, Miaomiao Wang, Xian Hu, Jihai Zhang, Qing Zhang, Congli Xu, Chunlin Long

**Affiliations:** 1 Key Laboratory of Ecology and Environment in Minority Areas (Minzu University of China), National Ethnic Affairs Commission of China, Beijing 100081, China; plantcqy@163.com (Q.C.);; 2 College of Life and Environmental Sciences, Minzu University of China, Beijing 100081, China; 3 College of Ethnology and Sociology, Minzu University of China, Beijing 100081, China; 4 Yunnan Gaoligongshan National Nature Reserve, Baoshan Bureau, Baoshan 678000, China; 5 Key Laboratory of Ethnomedicine (Minzu University of China), Ministry of Education, Beijing 100081, China; 6 Institute of National Security Studies, Minzu University of China, Beijing 100081, China

**Keywords:** *Disporopsis aspersa*, traditional knowledge, nutritional composition, medicinal food plants, ethnobotany

## Abstract

*Disporopsis aspersa* (Hua) Engl. ex K. Krause, locally known as *kucai* (bitter greens) or *yexiahua*, is a widely consumed wild vegetable and traditional herbal medicine in western Yunnan. Despite its local significance, its nutrient composition and bioactive properties have not been investigated. This study aims to determine the nutritional content and evaluate the antioxidant and anti-inflammatory activities of the aerial parts extracts of *D. aspersa*. The levels of protein, amino acids, vitamins, and minerals were measured and compared to those of common vegetables. The results showed that *D. aspersa* contains 16 amino acids, with a total content of up to 19.13 g/100 g, including 3.0 g/100 g of lysine. In vitro evaluations of its antioxidant and anti-inflammatory activities demonstrated that the ethanolic extract exhibited low cytotoxicity against mouse RAW 264.7 murine macrophages cell line at concentrations of 0–120 μg/mL. The IC_50_ for nitric oxide (NO) scavenging activity was 72.7 ± 7.43 μg/mL, showing dose dependence. Additionally, the ethanolic extract also exhibited ABTS^+^· scavenging capacity and total antioxidant capacity. These findings suggest that *D. aspersa* is rich in carbohydrates, fat, dietary fiber, and amino acids. It also contains various bioactive substances, supporting its traditional practices for both medicinal and dietary purposes by local people. *D. aspersa* has the potential to be developed into a novel anti-hypertensive food, nutraceutical, or dietary supplement in western Yunnan and neighboring regions, promoting local development.

## 1. Introduction

Diet plays a crucial role in influencing human health, as the 2022 Global Burden of Cardiovascular Disease project noted, particularly in relation to cardiovascular disease (CVD) [1]. Moreover, there are many overlaps between medicinal and dietary products, especially when it comes to medicinal and edible plants [2,3]. This connection not only emphasizes the importance of a balanced diet in disease prevention, but also highlights the significant market potential of food therapy as a holistic approach to health management. Therefore, wild edible plants (WEPs), which are rich in bioactive compounds, and natural therapies have gained significant interest due to their nutritional and medicinal properties [4,5]. Dietary therapy is becoming increasingly important in preventing and treating diseases and lifestyle disorders in both developing and developed countries [4,6]. Traditional knowledge (TK) of plants is the relationship between a community and the environment around it. Documentation of traditional knowledge is necessary for identifying WEPs to create production systems for sustainable use, commercialization, and conservation [5]. Based on TK, research into the nutrient value of wild vegetable resources and testing the bioactivity of their crude extracts will help us make better use of these resources.

*Disporopsis aspersa* (Hua) Engler ex K. Krause, a member of the family Asparagaceae, is a wild vegetable commonly consumed in the Yangtze River basin, including Sichuan, Guangxi, Hubei, Hunan, and Yunnan [7,8]. *Zhongyao Ziyuan Zhiyao of China* record that the rhizome of *D. aspersa* is used to treat rheumatic pain, rupture, and reducing internal inflammation [9]. *Zhongguo Minzuyao Zhiyao* listed it as an ethnomedicine of the Tujia people under the name *lluang shan qi*, with its rhizome being used to treat physical weakness, nourish the spleen and stomach, and alleviate food stagnation [10].

Even though it had preliminarily proved that *D. aspersa* has antifungal, neuritogenic, and anti-inflammatory activity [11], comprehensive evaluations of this species remain limited. Most previous studies have primarily focused on the anti-cancer effects and its ability to control potato late blight and cucumber downy mildew of its rhizome extracts. Song et al. and Nguyen et al. have verified the anti-cancer activity of the high-isoflavone compounds found in *D. aspersa* rhizome extracts, reporting its potential anti-cancer properties [12,13]. Zhu et al. conducted studies on the whole plant of *D. aspersa*, demonstrating that its crude extracts exhibit significant antibacterial activity against *Pseudoperonospora cubensis* and *Phytophthora infestans* [8]. Furthermore, other species within the genus *Disporopsis* also show broad pharmacological activities. Meng et al. screened for active components in *Disporopsis fuscopicta* that inhibit angiogenesis and confirmed that the isolated compounds effectively hinder the proliferation of human umbilical vein endothelial cells (ECV304) [14]. Tran et al. isolated two new spirostane glycosides from *Disporopsis longifolia* and evaluated their ability to inhibit nitric oxide (NO) production in lipopolysaccharide (LPS) activated RAW 264.7 cells, finding that both compounds exhibited strong anti-inflammatory activity [15]. This suggests that *D. aspersa* may possess undiscovered pharmacological potential. Known for its medicinal and edible properties, as well as its versatility and wide distribution, *D. aspersa* remains an enigmatic wild vegetable in the markets of the western Gaoligong Mountains in Yunnan. Mysteries persist regarding its cytotoxicity, nutritional composition, and the perceptions and management practices of local populations toward this resource.

This study aims to systematically evaluate the ethnobotanical relevance, nutritional composition, and pharmacological bioactivity of the aerial parts of *D. aspersa*, addressing its high seasonal demand and extensive traditional knowledge. Nutritional analysis will focus on quantifying proteins, lipids, carbohydrates, ash, amino acids, and minerals. Bioactivity assessments will include cytotoxicity, anti-inflammatory properties, and in vitro antioxidant activity of *D. aspersa* extracts.

## 2. Materials and Methods

### 2.1. Ethnobotanical Research

Ethnobotanical surveys were carried out three times in the western section of the Gaoligong Mountains in Yunnan from April 2023 to May 2024, and the entire study was conducted mainly in Longyang District and Tengchong City, Yunnan, China.

Ethnobotany field surveys included market surveys, semi-structured interviews, key informant interviews, and participatory observation [16,17,18]. In total, 18 informants (14 men and 4 women) between the age of 22 and 70 were selected using the snowballing method. The interviews included gathering data on traditional knowledge of *Disporopsis aspersa*, including the vernacular name, medicinal value, traditional uses, distribution, and growth, along with informants’ basic information (e.g., age, nationality, occupation). Pre-prepared images of the plant and its parts minimized misidentification. Collected information was organized, analyzed, and followed by field investigations to gather voucher specimens and materials.

In the process of investigation, the first recorded specimens, noting collection time, detailed location (e.g., latitude, longitude, altitude), and both local and Latin names, were collected. For the identification of plants, the voucher specimens were studied and compared with reference books (*Flora of China, Flora of Yunnan*) and online databases (https://www.iplant.cn/ and https://www.worldfloraonline.org/ (accessed on 11 November 2024)).

### 2.2. Preparation of Disporopsis aspersa

Samples of *D. aspersa* were selected and collected from markets in Longyang District and Tengchong City, Baoshan, Yunnan Province, in June 2023 and May 2024. Voucher specimens (specimen number: MUC-2024-13) were identified by Professor Long Chunlin and deposited in the herbarium at Minzu University of China, in Beijing.

The aerial parts were air-dried, ground, and sieved through a 40-mesh screen. Given its affinity for both water-soluble and lipid-soluble compounds, and combined with traditional knowledge usages of *D. aspersa*, 95% ethanol was selected as the extraction solvent to obtain an ethanolic extract (EE) [8,15,19]. Approximately 20 g of the ground material were weighed and extracted with 200 mL of 95% ethanol using Soxhlet extraction for 6 h [20]. The mixture was centrifuged at 12,000 r·min^−1^ for 10 min at 25 °C and the supernatant was collected. The residue underwent two additional extractions and the extracts were combined. The solvent was removed using an R-210 rotary evaporator (Büchi, CHN), and the resulting 2.35 g dried extract was freeze-dried at −80 °C. This process yielded the 95% ethanol extracts (EE), which were stored at −20 °C for future use.

### 2.3. Proximate Analysis

GB stands for the National Standards of the People’s Republic of China. These standards are formulated or approved by the Standardization Administration of China (SAC) and are designed to unify technical, managerial, and basic requirements across various fields nationwide. They are rigorously researched and validated, widely applied across industries to ensure the quality, safety, and reliability of products, services, and processes.

#### 2.3.1. Moisture Content

Percentages of moisture were determined by an electric constant temperature blower drying oven under 103 °C for 4 h to constant weight per the China national standards method (GB 5009.3-2016) [21,22].

#### 2.3.2. Ash Content

Ash was determined by direct analysis according to the China standards method (GB 5009.4-2016), the sample was fully carbonized to smokeless and weighed [21,23]. The dry sample was then placed in the energy-saving box-type furnace at 550 °C for 4 h, removed, and placed into a desiccator for 30 min after cooling down to 200 °C. The whole process was repeated until to a constant weight.

#### 2.3.3. Fat Content

Total fat content was determined by solvent extraction method according to the China standards method (GB 5009.6-2016) [24,25].

#### 2.3.4. Total Dietary Fiber Content

Dietary content was determined according to the China standards method (GB 5009.88-2014) [26,27]. Take the dried samples and crush them repeatedly until they are completely sieved. After the protein and starch were removed by the enzymolysis of heat-stabilized α-amylase, protease, and glucosidase, the dried sample was precipitated by ethanol, pumped and filtered, the residue was washed with ethanol and acetone, dried and weighed, and the total dietary fiber residue was obtained.

#### 2.3.5. Crude Protein

Protein content was determined by Kjeldahl nitrogen using the China standards method (GB 5009.5-2016) [28]. The percentage of crude protein was estimated as the total nitrogen content multiplied by the conversion factor 6.25.

#### 2.3.6. Carbohydrate and Energy Content

Total carbohydrates were calculated by subtracting the total percentage of other components (saccharides, starch, and dietary fiber) from 100 according to the China standards method (GB/Z 21922-2008) [29,30].

Carbohydrates can be calculated using the following formula:

(1) Carbohydrates=100−Protein−Fat−Moisture−Ash−Dietary Fiber,

Energy can be calculated using the following formula:

(2) Energy=Protein×17+Carbohydrates×17+Fat×37+Dietary Fiber×8,

### 2.4. Mineral Composition

The mineral elements were determined using an inductively coupled plasma mass spectrometer (ICP-MS) (A1-IE-2855, PONY, Beijing, China). For quantification, an external standard method was used, where the intensity ratio of the target element’s mass spectrometric signal to that of an internal standard element was directly proportional to the concentration of the target element. Potassium, phosphorus, sodium, iron, manganese, magnesium, calcium, copper, and zinc were determined using GB 5009.268-2016 (the second method) [31], while Se was determined using GB 5009.93-2017 (the first method) [32,33].

### 2.5. Amino Acid Composition

Amino acids were measured according to the method GB 5009.124-2016 by Amino acid Analyzer (A1-IE-2855, PONY) [24,34]. Data were expressed as milligrams of amino acid per 100 g of *Disporopsis aspersa*.

### 2.6. Vitamin Composition

Vitamin E was determined using reversed-phase high-performance liquid chromatography (RP-HPLC) (A1-IE-4812, PONY) according to GB 5009.82-2016 [35,36]. Vitamin B_1_ and vitamin B_2_ was determined using high-performance liquid chromatography (HPLC), according to GB 5009.84-2016 and GB 5009.85-2016, respectively. Vitamin B_6_ was determined using microbiological assay according to GB 5009.154-2016 (the second assay) [37,38,39,40]. Vitamin K_1_ was determined using High-Performance Liquid Chromatography with Fluorescence Detection (HPLC-FLD) according to GB 5009.158-2016 [41].

### 2.7. Antioxidant Activity

DPPH· scavenging capacity was assessed using a DPPH Antioxidant Assay Kit (Dojindo, Shanghai, China), while ABTS^+·^ scavenging capacity and total antioxidant capacity were measured using ABTS and FRAP Assay Kits (Beyotime, Shanghai, China) [42,43]. The ABTS rapid method evaluates antioxidant capacity by measuring the ability to inhibit ABTS^+·^ formation, whereas the FRAP method assesses the ability of antioxidants to reduce Fe^3+^-TPTZ under acidic conditions. Both methods utilize Trolox as a standard. Trolox, a water-soluble vitamin E analogue, has antioxidant activity similar to that of vitamin E. DPPH and ABTS^+·^ scavenging capacities are expressed in terms of Trolox equivalents, while total antioxidant capacity is expressed as Fe^2+^ equivalents.

### 2.8. Anti-Inflammatory Activity

#### 2.8.1. Cell Culture

RAW 264.7 cell lines are frequently used to evaluate anti-inflammatory activity of plant extracts by measuring inhibition of lipopolysaccharide-induced (LPS-induced) nitric oxide (NO) production [44]. Therefore, this study selected mouse myoblast cell line RAW 264.7 (Cell Resource Center, Beijing, China). RAW 264.7 cells were cultured in DMEM (Gibco, Shanghai, China) high-glucose complete medium containing 10% (*v*/*v*) Fetal Bovine Serum (HyClone, Shanghai, China) and 1% penicillin-streptomycin at 37 °C in a 5% CO_2_ atmosphere.

#### 2.8.2. Cell Viability

To access the potential cytotoxicity and anti-inflammatory activity of the EE, RAW 264.7 cells were pre-induced with LPS for 18 h [45,46], followed by different concentrations of crude extract treatments. The impact of the EE on RAW 264.7 cells viability was detected using the Cell Counting Kit-8 (CCK-8) (Dojindo, China). After the cells adhered, the EE at varying concentrations were applied for 18 h. Then, suck out the supernatant and add 100 µL of 10% CCK-8 solution to cultures, followed by incubation at 37 °C for 1 h. The OD at 450 nm was measured by a Microplate Reader (Epoch TM, San Jose, CA, USA). According to the OD_450_ values, the cell viability was calculated as follows:

(3) $$Cell\;viability(\%)=\frac{AS-ASC}{AC-ASC}\times 100\%$$

where AS: absorbance of the sample, AC: absorbance of the control, and ASC: absorbance of solvent control.

#### 2.8.3. Determination of NO Production

Cells (4 × 10^5^ cells/well in 2000 μL of medium) were cultured overnight in a six-well plate. After cell adhesion, the varying concentration EEs were applied with or without 1 μg·mL^−1^ LPS to induce an inflammatory response. Blank controls were served as no LPS and no EE treatment. After 18 h incubation, the culture supernatant from each well was collected to measure the NO levels using an NO detection kit (Sigma-Aldrich, Saint Louis, MO, USA) to assess the level of inflammation in the culture medium.

### 2.9. Statistical Analysis

The results are presented as mean ± standard deviation (SD), with all experiments performed in triplicate (*n* = 3) by GraphPad Prism 8.3.0. Data were analyzed using one-way analysis of variance (ANOVA), followed by Duncan’s multiple range test.

## 3. Results and Discussion

### 3.1. Ethnobotanical Knowledge

**Figure 1:**
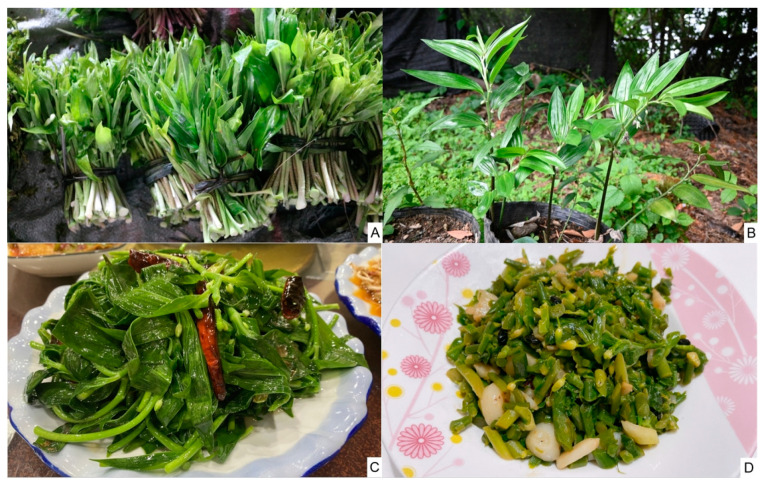
The plant of *Disporopsis aspersa* in the study area. (**A**) Aboveground part sold on market; (**B**) mature leaves; (**C**,**D**) stir-fried dish of *D. aspersa* showing flower buds (photographed credit: the authors).

*Disporopsis aspersa* (Hua) Engler ex K. Krause (Asparagaceae) is a herbaceous plant with both edible and medicinal properties (Figure 1). Wild *D. aspersa* in western Yunnan typically grows at altitudes of approximately 3000 m, primarily beneath bamboo forests or along ravines, often coexisting with plants from the genus *Allium*, *Trillium*, and *Rhododendron*. The local harvest season for *D. aspersa* occurs during its flowering period in April and May, when locals selectively harvest young leaves, tender stems and flower buds, leaving the mature parts untouched. Each area is harvested only once per season. Regarding sustainable harvesting practices, locals report that *D. aspersa* thrives with regular harvesting and does not face the risk of overharvesting. Specifically, only young shoots taller than 10 cm are harvested, and hardy parts of the plants are left undisturbed. This approach reflects the ecological awareness and plant conservation in local communities.

Locals in the western Gaoligong Mountains refer to *D. aspersa* by several names based on its various morphological features. The name *Zhu-jie-cai* is derived from its leaf shape, which resembles bamboo leaves. *Niu-wei-ba-cai* refers to its young shoots, which resemble an ox tail. *Zhu-ye-cai* is based on its growth near bamboo groves. *Ku-cai* reflects its characteristic bitter taste when consumed. Its tender leaves can be served in salad, stir-fried, or used to make soups. It is widely consumed by both the Han people and Lisu people in the area.

Ethnobotanical surveys show that *D. aspersa* is widely recognized by local residents for its dual medicinal and dietary uses (Table 1). The entire plant is consumed as food or used in traditional medicine, with applications including lowering blood pressure, treating coughs, cooling and detoxifying the body, and nourishing the lungs and stomach. The tender leaves are often eaten raw, stir-fried, or cooked in soups, providing a palatable flavor, while the roots are commonly stir-fried, stewed, steamed, or cooked with chicken, imparting a slightly bitter taste. Additionally, *D. aspersa* is regarded as an excellent topical remedy for cuts and bruises, known locally as *Ye-xia-hua* (flower under the leaves). In this usage, crushed leaves are applied to wounds and usually changed daily, promoting wound healing and suggesting antibacterial and anti-inflammatory properties. Traditional knowledge suggests that *D. aspersa* has potential anti-inflammatory and antioxidant properties, and significant nutritional value.

Different linguistic groups have varying usages: the Han people primarily consume it as food, while the Lisu people apply it externally for wounds. Over time, the Lisu people have also begun to consume it as a vegetable, reflecting cultural exchange. This diversity of uses highlights its dual role as both food and medicine. During the course of our research, local residents frequently compared *D. aspersa* to other medicinal plants such as *Panax notoginseng* (sanqi), *Basella alba*, and *Polygonatum* species, indicating its potential anti-inflammatory effects and medicinal efficacy. In the research regions, traditional knowledge of *D. aspersa* has been passed down through generations. Additionally, due to its aesthetically pleasing appearance, *D. aspersa* is often grown as an ornamental plant.

Since the establishment of the Gaoligong Mountains Nature Reserve in the 1980s, to reduce the difficulty of harvesting, the locals transplant *D. aspersa* into household gardens at lower elevations, around 1500 m. It can be cultivated near large trees or water sources, requiring only natural rainfall for growth, with minimal additional care. Although some individuals believe that wild *D. aspersa* has a superior aftertaste compared to cultivated varieties, the market prices of both are comparable, indicating the potential for sustainable development. Given its natural distribution in the Yangtze River basin, including Sichuan, Hubei, Hunan, and Yunnan, *D. aspersa* deserves to be promoted as a seasonal specialty vegetable.

### 3.2. Nutrient Compositions

#### 3.2.1. Proximate Analysis

The general nutrient composition and dietary fiber content of *D. aspersa* are summarized in Table 2. Given that Chinese cabbage (*Brassica rapa*) and spinach (*Spinacia oleracea*) are among the most commonly consumed leafy vegetables globally, they were used as benchmarks for comparison. Their nutrient composition comes from *China Food Composition Tables (Standard Edition)* and USDA National Nutrient Database (https://fdc.nal.usda.gov (accessed on 11 November 2024)) [47]. Carbohydrates (49 g/100 g) are the primary component of *D. aspersa*, followed by total dietary fiber (37.73 g/100 g) and protein (27.13 g/100 g). Although the fat content is the lowest at 5.60 g/100 g, it is still significantly higher than that of Chinese cabbage and spinach. Except for carbohydrates, *D. aspersa* surpasses both reference vegetables in all other nutritional components.

Dietary fiber (DF), derived from the edible parts of plants, is a carbohydrate-like substance that undergoes fermentation and decomposition by the gut microbiota in the colon. Insufficient DF intake is a modifiable risk factor for chronic disease, making increased DF intake an effective prevention strategy [48,49]. Legumes are generally considered to be high in dietary fiber, with common bean seeds containing 16.21 to 24.50 g/100 g of total DF, which is lower than the 37.73 g/100 g found in *D. aspersa* [50,51]. As a functional nutrient, DF exerts significant physiological effects, such as shortening transit time, delaying gastric emptying, promoting intestinal motility, modulating gut microbiota, and improving metabolic disorders and immune system function. These effects support weight management for individuals with obesity, as well as reducing the incidence of cardiovascular disease (CVD) and colorectal cancer (CRC) [52,53]. Therefore, *D. aspersa* has the potential to be considered a functional food that meets modern health dietary requirements, offering benefits not only for improving dietary structures in patients but also for disease prevention in the general population.

#### 3.2.2. Mineral Elements and Vitamins

Ten minerals have been selected for detection in *D. aspersa*. Five of them are essential trace elements for humans, including iron (Fe), zinc (Zn), copper (Cu), chromium (Cr), and magnesium (Mg), as shown in Table 2. Notably, *D. aspersa* contains particularly high levels of potassium (K) and phosphorus (P), significantly exceeding those found in spinach and Chinese cabbage. The K content in *D. aspersa* is 4803.33 mg/100 g, which is substantially higher than that of K-rich vegetables such as spinach (919 mg/100 g). Conversely, its sodium (Na) content is relatively low at 5.46 mg/100 g, especially when compared to spinach (242 mg/100 g), classifying *D. aspersa* as a low-sodium, high-potassium vegetable, making it particularly beneficial for individuals with hypertension. In comparison to Fe-rich wild or cultivated vegetables like spinach, *Diplazium esculentum* (23.7 mg/100 g) and *Maianthemum atropurpureum* (20.3 mg/100 g), *D. aspersa* exhibits comparable levels of Fe (29.67 mg/100 g) and Mg (197.67 mg/100 g) [6]. Furthermore, the zinc content in *D. aspersa* (5.62 mg/100 g) is slightly higher than that found in well-known zinc-rich vegetables like *Brasenia schreberi* (1.53–2.88 mg/100 g) and spinach (3.91 mg/100 g) [54].

Recent studies have identified the interaction between excessive Na and K deficiency as key environmental factors in the pathogenesis of hypertension and cardiovascular disease (CVD) [53], with both Na and K playing significant roles in this process. This result nicely explains traditional applications that consuming *D. aspersa* could lower blood pressure. Increasing K intake through fresh fruits and vegetables can mitigate the detrimental effects of excessive Na, making K supplementation an effective strategy for primary prevention and treatment of hypertension and CVD, which may be more feasible than sodium restriction [55]. Moreover, the effective supplementation of K can rapidly restore energy levels, making *D. aspersa* a particularly important vegetable resource for local agricultural labor. Thus, *D. aspersa* serves as a valuable dietary source of K, P, and other nutrients, aiding individuals deficient in these essential elements in maintaining health.

The vitamin E content in *D. aspersa* is significantly higher than that in spinach and Chinese cabbage. It exceeds that of commonly recognized vitamin E-rich vegetables such as asparagus (*Asparagus officinalis*, 1.2–1.5 mg/100 g) [56] and beetroot (1.81 mg/100 g). Additionally, the contents of water-soluble vitamins B_1_, B_2_, and B_6_ are relatively high. The high level of vitamin K_1_ and vitamin B_6_ in *D. aspersa* are beneficial for cardiovascular health, as vitamin K_1_ is essential for blood clotting, playing a key role in the synthesis of several clotting factors. This also indicates that *D. aspersa* may help prevent cardiovascular diseases. Furthermore, given the local practices of stir-frying, boiling, and using *D. aspersa* in salads, its consumption could help prevent vitamin and trace element deficiencies, thereby supporting normal physiological functions.

#### 3.2.3. Amino Acids

The 16 amino acids were identified in *D. aspersa* and the results are listed in Table 3, with a total amino acid content reaching 19.13 g/100 g. Seven of these are essential amino acids (EAAs), totaling 8.11 g/100 g, representing 42.37% of the total and 73.52% of the non-essential amino acids (NAAs). According to the ideal model proposed by the World Health Organization (WHO) and the Food and Agriculture Organization (FAO), high-quality protein should have an EAA to total amino acid (EAA/TAA) ratio of approximately 40%, and an EAA to NAA (EAA/NAA) ratio exceeding 60% [52,57]. Based on these criterion, *D. aspersa* qualifies as a high-quality protein source, with EAA/TAA and EAA/NAA ratios surpassing Chinese cabbage and spinach.

Additionally, *D. aspersa* exhibits the highest concentration of lysine (3.00 g/100 g), followed by glutamic acid (2.27 g/100 g), aspartic acid (1.70 g/100 g), histidine (1.60 g/100 g), and leucine (1.41 g/100 g). Lysine, an essential amino acid that cannot be synthesized by the human body, must be acquired through dietary sources. It promotes calcium absorption and utilization, aids in protein synthesis and tissue repair, and supports bone and muscle growth. Furthermore, lysine plays a crucial role in the production of various important substances in the body, including hormones, enzymes, and antibodies, which are vital for maintaining normal physiological functions [58,59]. Glutamic acid, the primary excitatory neurotransmitter in the central nervous system, is essential for brain function, playing a role in learning, memory, and cognition. Additionally, it also contributes to ammonia production in the kidneys, helping maintain the body’s acid-base balance [60].

The findings indicate that *D. aspersa* is an excellent source of amino acids, providing nearly all the amino acids required by humans. The high content of lysine, aspartic acid, and glutamic acid in *D. aspersa* contributes to its umami flavor, while histidine and alanine contribute to its sweetness [61,62]. The total content of these five flavor-enhancing amino acids is 9.86 g/100 g, accounting for 51.54% of the total amino acids, giving *D. aspersa* its characteristic sweet aftertaste.

### 3.3. Antioxidant Activity

Given its affinity for both water-soluble and lipid-soluble compounds, 95% ethanol was selected as the extraction solvent to obtain an ethanolic extract (EE) [8,19]. The ABTS^+·^ scavenging capacity of the EE of *D. aspersa* was measured at 55.11 ± 0.31 μmol Trolox·g^−1^, and its total antioxidant capacity was 57.97 ± 1.04 µmol (Fe^2+^)·g^−1^. The trends shown in Table 4 in all three antioxidant capacity evaluation indicators were consistent, indicating a high level of reliability in the antioxidant assessment results.

*D. aspersa* showed potential activity in scavenging hydroxyl radicals at various concentrations, similar to that of essential oil of *Disporopsis fuscopicta*, which exhibited a scavenging capacity with 0.04 ± 0.008 mmol (Fe^2+^)·g^−1^ [63]. According to Li et al. [64], rutin, luteolin, quercetin, and betulinic acid, extracted from the ethyl acetate extract of *D. fuscopicta*, were all proven to possess strong antioxidant activity. Flavonoids are generally recognized for their great antioxidant capacity [65]. Previous research has isolated a variety of bioactive substances such as rutin, quercetin, and salicylate from *D. aspersa*, suggesting that flavonoids and phenolic acids may be the main active components responsible for its antioxidant activity [8,13].

### 3.4. Anti-Inflammatory Activity

**Figure 2:**
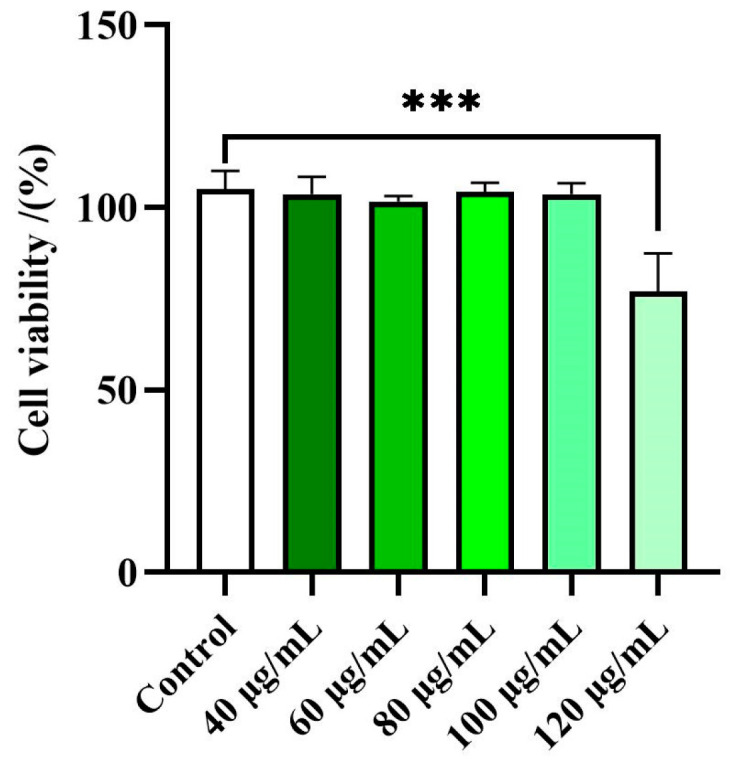
Effects of the EE of *Disporopsis aspersa* on RAW 264.7 cell viability. Results are expressed as percentage of cell viability relative to untreated control cells (Control). Each bar shows mean ± SD of three independent experiments performed in triplicate (*** *p* < 0.01 compared to Control).

Preliminary experiments to evaluate the effects of the EE of *Disporopsis aspersa* on cell viability were carried out using a CCK-8 test. RAW 264.7 macrophage cells were treated with the EE at different concentrations (40–120 μg/mL) for 18 h. It is considered that a cell viability rate exceeding 80% indicates that the sample has no cytotoxic effect on the tested cells [66]. As illustrated in Figure 2, the 95% ethanol extracts (EE) exhibited no significant cytotoxicity towards RAW 264.7 cells at concentration below 120 μg/mL. Consequently, further studies on the EE were conducted, keeping the concentration below 120 µg/mL.

The anti-inflammatory effects of the EE were investigated by measuring nitric oxide (NO) production using the Griess reagent. NO is a signaling molecule that plays a crucial role in the progression of inflammation. Therefore, NO inhibitors are considered potential therapeutic targets for therapy anti-inflammatory [67].

**Figure 3:**
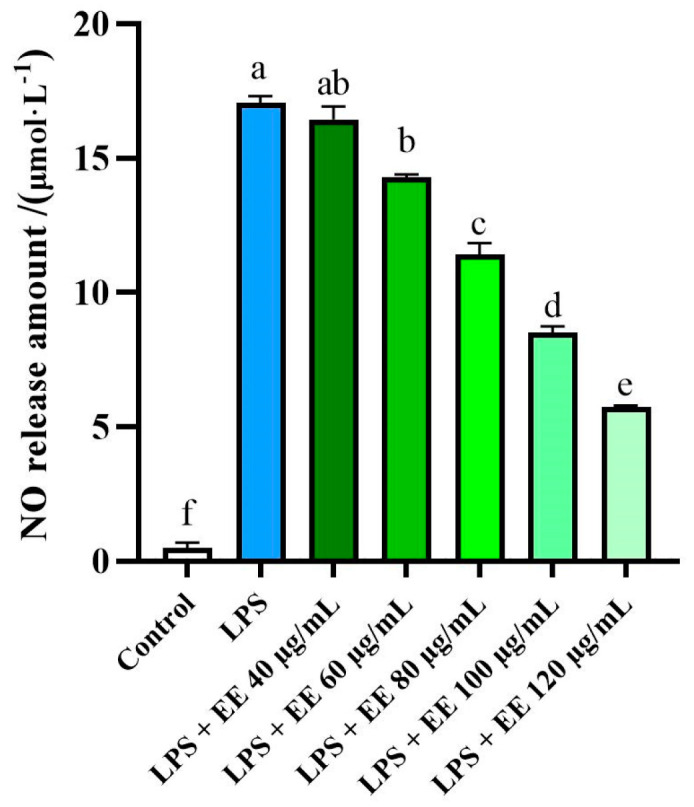
Effects of the EE on RAW 264.7 cells NO production induced by LPS, expressed as mean ± standard deviation (*n* = 3). Bars with different letters indicate significant difference (*p* < 0.05).

As illustrated in Figure 3, the EE exhibited significant NO inhibitory effect (IC_50_ = 72.7 ± 7.43 μg/mL), demonstrating a certain dose-dependence. Lipopolysaccharide (LPS) significantly induces the production of NO compared to control cells, confirming the successful establishment of the LPS-induced inflammatory model in RAW 264.7 cells. As the concentration of the *D. aspersa* ethanol extract increased, NO production in RAW264.7 cells gradually decreased. These results indicate that the EE has anti-inflammatory properties, mediated by the inhibition of NO production.

Consistent with previous studies, Gang et al. found that 2-isopropyl-5-methyphenyl 2-(naphthalen-1-yl)acetate and (3*R*,4*S*)-3,4-dihydroxy-3-methyldihydrofuran-2(3*H*)-one isolated from *D. aspersa* exhibit moderate NO inhibition in LPS-induced BV-2 microglial cells [7]. Furthermore, Song et al. revealed that 3-(4′-hydroxy-benzyl)-5, 7-dihydroxy-6-methyl-8-methoxyl-chroman-4-one isolated from *D. aspersa* significantly inhibit SW620 and MDA-MB-231 cells, with IC_50_ values of 32.5 μg/mL and 42.7 μg/mL, respectively [12]. Nguyen et al. also showed that several flavonoids display cytotoxicity across six cancer cell lines [13]. Flavonoids, phenolic acids, and steroids are widely recognized for their anti-inflammatory and antioxidant properties, and these compounds are more easily extracted from ethanolic extracts [65,68,69]. The flavonoids isolated from *D. aspersa* provide a theoretical basis for their potential medicinal applications.

Other *Disporopsis* species also demonstrate anti-inflammatory potential. Meng et al. identified angiogenesis inhibitors in *D. fuscopicta*, effective against ECV304 cells [14]. Thu Ha et al. isolated two spirostanol glycosides from *D. longifolia* that strongly inhibit NO production in LPS-activated RAW 264.7 cells, with IC_50_ values of 26.6 ± 1.9 μM and 24.5 ± 2.5 μM [15]. According to this study and previous literature, *D. aspersa* has shown good potential for anti-inflammatory and anti-tumor effects, as well as a certain antioxidant capacity. This provides a scientific basis for the traditional use of healing trauma in ethnobotanical surveys, indicating that this plant may have the potential to support further exploration for natural anti-inflammatory agents.

## 4. Conclusions

In the western region of the Gaoligong Mountains in Yunnan, the local people have rich traditional knowledge about and practical applications for *Disporopsis aspersa*. However, the true value of this medicinal food plant remains underestimated due to insufficient evaluation. Understanding the nutritional and medicinal properties of this underutilized vegetable can promote its conservation and broader use in human diets as a valuable source of nutrition and anti-inflammatory agents.

*D. aspersa*, containing higher levels of calcium, magnesium, potassium, vitamins E, vitamins B_2_, and vitamins B_6_, as well as amino acids like Val, Leu and Lys, is a high-energy wild vegetable and an important source of high-quality protein. It also provides substantial dietary fiber and essential minerals, supporting diverse diets and promoting healthier eating patterns while reducing carbon footprints.

In addition to its nutritional benefits, *D. aspersa* exhibits potential anti-hypertensive, anti-inflammatory, and antioxidant properties. As a commonly consumed wild edible herb, its medicinal effects influence local dietary choices. Its high vitamin E and dietary fiber content, combined with a low-sodium and high-potassium profile, contribute to its role in regulating blood pressure, benefiting individuals with cardiovascular diseases. Furthermore, *D. aspersa* also exhibits strong anti-inflammatory and moderate antioxidant activities, supporting its use in wound healing and inflammation control. Based on this study and previous literature, the abundant flavonoids, phenolic acids, and steroids in *D. aspersa* may contribute to its remarkable anti-inflammatory properties. However, further research is needed to clarify the relationship between these metabolites and their bioactivities.

The genus *Disporopsis*, to which *Disporopsis aspersa* belongs, is primarily distributed in Southeast Asia and has a wide geographical range. This study scientifically validates the traditional knowledge of *D. aspersa*, offering insights for research on *Disporopsis* species in other regions and providing clues for the development and utilization of edible plants in the Asparagaceae family by other countries. This would not only restore cultural and food heritage but also create sustainable production and consumption alternatives.

## Figures and Tables

**Table 1:** The vernacular names of *Disporopsis aspersa* in different linguistic groups.

Vernacular Name	Linguistic Group	Methods of Usage
*Zhu-jie-cai*	Han, Lisu	Tender leaves are often eaten raw, stir-fried, or cooked in soups; the roots are commonly stir-fried, stewed, steamed, or cooked with chicken.
*Ku-cai*	Han, Lisu, Bai, Zhuang	
*Niu-wei-ba-cai*	Han	
*Ye-xia-hua*	Lisu	The aboveground ground portion is crushed and used as a trauma medicine.

**Table 2:** Proximate composition, mineral, and vitamin content of 100 g *Disporopsis aspersa* and other two well-known edible greens.

	Unit	*Disporopsis aspersa*	*Brassica rapa*	*Spinacia oleracea*
Protein	g	27.13	1.6	2.6
Carbohydrate	g	49	3.4	75.7
Fat	g	5.60	0.2	0.6
Total dietary fiber	g	37.73	0.9	1.7
Energy	kJ	1501	82	1290
Mineral content				
Sodium (Na)	mg	5.46	68.90	242.00
Selenium (Se)	mg	0.02	Nd	Nd
Copper (Cu)	mg	1.08	0.06	2.08
Zinc (Zn)	mg	5.62	0.46	3.91
Iron (Fe)	mg	29.67	0.80	25.90
Calcium (Ca)	mg	286.67	57.00	411.00
Magnesium (Mg)	mg	197.67	12.00	183.00
Potassium (K)	mg	4803.33	134.00	919.00
Manganese (Mn)	mg	3.15	0.19	1.61
Phosphorus (P)	mg	726.00	33.00	222.00
Vitamins				
Vitamin E	mg	16.60	0.36	7.73
Vitamin K_1_	mg	0.22	0.48	0.3
Vitamin B_1_	mg	0.15	0.04 ^1^	0.08 ^1^
Vitamin B_2_	mg	0.40	0.03 ^1^	0.19 ^1^
Vitamin B_6_	mg	0.33	0.19 ^1^	0.20 ^1^

^1^ Data from the USDA National Nutrient Database.

**Table 3:** Amino acids of *Disporopsis aspersa* compared with other two well-known edible greens.

	Unit	*Disporopsis aspersa*	*Brassica rapa*	*Spinacia oleracea*
Thr	G	0.80	0.04	0.11
Val	G	1.11	0.05	0.12
Met	G	0.04	0.01	0.02
Ile	G	0.81	0.04	0.10
Leu	G	1.41	0.06	0.18
Phe	G	0.92	0.04	0.11
Lys	G	3.00	0.05	0.15
EAA	G	8.11	0.29	0.79
Asp	G	1.70	0.11	0.23
Ser	G	0.84	0.05	0.10
Glu	G	2.27	0.37	0.33
Pro	G	0.76	0.04	0.10
Gly	G	0.90	0.04	0.14
Ala	G	1.29	0.07	0.13
Tyr	G	0.52	0.03	0.08
His	G	1.60	0.02	0.06
Arg	G	1.15	0.06	0.13
NAA	G	11.03	0.77	1.30
TAA	G	19.13	1.07	2.09
EAA/TAA	%	42.37%	27.39%	37.70%
EAA/NAA	%	73.52%	37.73%	60.51%
Lys/TAA	%	15.70%	4.78%	7.02%

**Table 4:** Antioxidant capacity of the EE of *Disporopsis aspersa*.

Species	DPPH· Scavenging Ability (μmol Trolox·g^−1^)	ABTS^+·^ Scavenging Ability (μmol Trolox·g^−1^)	Total Antioxidant Ability (μmol FeSO_4_·g^−1^)
*D. aspersa*	5.49 ± 0.01	55.11 ± 0.31	57.97 ± 1.04

## Data Availability

The original contributions presented in this study are included in the article. Further inquiries can be directed to the corresponding authors.
